# Self-reported health status and access to health services in a sample of prisoners in Italy

**DOI:** 10.1186/1471-2458-11-529

**Published:** 2011-07-04

**Authors:** Carmelo GA Nobile, Domenico Flotta, Gaetano Nicotera, Claudia Pileggi, Italo F Angelillo

**Affiliations:** 1 Chair of Hygiene, Medical School, University of Catanzaro "Magna Græcia", Catanzaro, Italy; 2 Department of Experimental Medicine, Second University of Naples, Naples, Italy; 3 At the time of this study he was with the Chair of Hygiene, Medical School, University of Catanzaro ''Magna Græcia'', Catanzaro, Italy

## Abstract

**Background:**

Self-reported health status in underserved population of prisoners has not been extensively explored. The purposes of this cross-sectional study were to assess self-reported health, quality of life, and access to health services in a sample of male prisoners of Italy.

**Methods:**

A total of 908 prisoners received a self-administered anonymous questionnaire pertaining on demographic and detention characteristics, self-reported health status and quality of life, access to health services, lifestyles, and participation to preventive, social, and rehabilitation programs. A total of 650 prisoners agreed to participate in the study and returned the questionnaire.

**Results:**

Respectively, 31.6% and 43.5% of prisoners reported a poor perceived health status and a poor quality of life, and 60% admitted that their health was worsened or greatly worsened during the prison stay. Older age, lower education, psychiatric disorders, self-reported health problems on prison entry, and suicide attempts within prison were significantly associated with a perceived worse health status. At the time of the questionnaire delivery, 30% of the prisoners self-reported a health problem present on prison entry and 82% present at the time of the survey. Most frequently reported health problems included dental health problems, arthritis or joint pain, eye problems, gastrointestinal diseases, emotional problems, and high blood pressure. On average, prisoners encountered general practitioners six times during the previous year, and the frequency of medical encounters was significantly associated with older age, sentenced prisoners, psychiatric disorders, and self-reported health problems on prison entry.

**Conclusions:**

The findings suggest that prisoners have a perceived poor health status, specific care needs and health promotion programs are seldom offered. Programs for correction of risk behaviour and prevention of long-term effects of incarceration on prisoners' health are strongly needed.

## Background

Prisoners' health represent one of the major challenge for public health and they have often the greatest needs since poor socio-economic conditions are associated with multiple health risks and greater morbidity and mortality [1-3]. Thus, prisoners tend to show high morbidity rate at admission, especially for chronic diseases, mental illnesses, infectious diseases, and sexually transmitted diseases (STDs) [4-8]. These highly vulnerable individuals have generally poor access to health services outside the prison and, once entered into penitentiary centres, most of them will return in the community after a short period of detention [9]. Hence, the prison may represent an opportunity to assist public health in a cost-effective manner, that benefits the society at whole, through prevention and treatment of diseases and correction of risk behaviours in this subgroup [9]. For instance, it has been established that prisoners are at increased risk of death after release from prison, mainly in the first two weeks, and important risk factors that former prisoners face are drug overdose, cardiovascular disease, homicide, and suicide [10-12]. The provision of specific interventions aimed at risk reduction while prisoners are in custody may prevent the high rate of recidivism and morbidity and could facilitate their adjustment in the community [10,11,13].

Self-rated health is commonly reported as a subjective indicator, as a strong predictor of longer-term morbidity and mortality, and as a method to identify high-risk groups with health needs [14,15]. Studies assessing self-rated health and chronic conditions in prisoners showed a perceived poor health status and high morbidity, especially in comparison with the general population [2,8,9,16]. Since similar research has not been conducted in Italy, this survey was carried out with the aims of assessing their self-rated health, the utilization of prisons health services, and the role of demographic and selected characteristics related to imprisonment status on the outcomes of interest among a sample of prisoners in Italy.

## Methods

A cross-sectional survey was conducted between February and December 2005 in Calabria, South of Italy, through a self-administered questionnaire on a population of male prisoners. Out of the 10 penal institutions located in the area, that can take into custody 2,347 prisoners [17], four institutions, for 1,085 potential participants including 463 in Catanzaro, 242 in Vibo Valentia, 209 in Rossano, and 171 in Castrovillari, were randomly selected. The whole prisoner population able to read Italian from the selected institutions was invited to participate. A total of 908 prisoners, 83.7% of the total eligible population, were enrolled in the study. Participation in the survey was voluntary, and prisoners' answers were anonymous and confidential.

A pretest was carried out on a convenience sample of 30 prisoners from the target population, in order to evaluate clarity, comprehensiveness, and acceptability of the questionnaire [18]. Feedback was incorporated into the questionnaire prior to the initial delivering.

In order to increase the participation, Prisons Medical Officers, who had been previously trained, were involved in handing out and in collecting self-administered questionnaires and were advised to respond to prisoners' queries only about the procedure and to guarantee the independent completion of the questionnaire.

The questionnaire included an introduction aimed at detailing the objectives of the study and at guaranteeing anonymity and confidentiality of gathered data, and four further sections pertaining demographic and detention characteristics, self-reported health status and access to health services, lifestyles, with special focus on smoking habits and substance abuse, and participation in preventive, social, and rehabilitation programs. The section on demographic and detention characteristics focused on age, marital status, number of children, education attainment, employment status at prison entry, dealing with first incarceration, overall time spent in prison, stage of adjudication, and length of sentence. Self-reported health status and access to health care services were investigated using a set of 11 items appraising self-rated health and quality of life, change in self-reported health status following prison entry, frequency and reasons for accessing to health care services within prison, self-reported health problems at prison entry, self-reported current health problems, concerns about acquiring STDs, experiencing negative feelings, and suicide attempts both outside and inside prison. The response levels for items assessing perceived health and quality of life were arranged using "poor", "good", and "very good", change the perceived current health status compared with the status preceding prison entry was explored using a Likert-like scale with five-point response set ranging from "greatly worsened" to "greatly improved". Access to health care services were explored using a "yes" or "no" format, self-reported current symptoms or diseases and reasons for medical encounters were explored using a half-open item, while five-point ordered-category items ranging from "never" to "very often" were used to detail the frequency of arranging medical encounters with general practitioners and the frequency of experiencing negative feelings; concerns on acquiring STDs were evaluated using a discrete visual analog scale with responses ranging from 1 (perceived low risk) to 10 (perceived high risk). The lifestyle section comprised six items investigating smoking habits and substances addiction: smoking status ("never smoker", "former smoker", and "current smoker"), daily number of cigarettes smoked by current smokers; lifetime history of drugs use, both outside and inside prison, was appraised using items arranged on "yes" or "no" levels, whereas the type of substances was asked using half-open items. The section on preventive programs aimed to elicit prisoners participation to health promotion and rehabilitative programs provided in prison on tobacco smoking, alcohol abuse, STDs, and unhealthy nutrition habit was based on a set of items with "yes" or "no" response levels. Participation in social and rehabilitation programs was investigated by asking prisoners whether they had attended to vocational training programs ("yes" or "no"), and then offenders were asked to deem usefulness of such training programs in facilitating their reintegration into work and social life ("useful", "not useful", and "uncertain") and employment expectation once outside prison ("unemployed", "previous job", "new job", and "uncertain").

Questions on self-rated health and health modification during the period of incarceration were derived mainly from the SF-36 questionnaire, while questions on health service use and other items were drawn out from the literature. Items had been modified, if necessary, by taking into account feedback from the pretest.

The study was approved by the Institutional Ethical Committee ("Mater Domini" Hospital of Catanzaro, Italy, study number 2005/47).

## Statistical analysis

Two separate multiple stepwise logistic regression with backwards elimination were used to identify major independent predictors to the following primary outcomes of interest: self-rated health status (Model 1) and access to health services within prison (Model 2). For the purposes of analysis, the outcome variables originally consisting of three categories in the logistic analysis were collapsed into two levels. In Model 1, respondents were divided into those who rated as "poor" their health (recoded as 1) versus those who rated their health as "good/very good" (recoded as 0); in Model 2, respondents were divided into those who consulted "sometimes/often/very often" general practitioners (recoded as 1) versus those who consulted them "never/rarely" (recoded as 0). In all models, the independent variables included were the following: age (continuous, in years), marital status (unmarried/separated/divorced/widowed = 0, married = 1), education level (no formal education = 0, compulsory education or more = 1), employment status before entering prison (unemployed = 0, employed = 1), smoking habit (past/never smoker = 0, current smoker = 1), having ever used drugs (no = 0, yes = 1), first time incarceration (no = 0, yes = 1), time served in prison (continuous, in years), detention status (categorical, awaiting trial = 0, awaiting sentence = 1, serving sentence = 2), self-reported health problems at prison entry (no = 0, yes = 1), self-reported current health problems (no = 0, yes = 1), negative mood (never/rarely = 0, sometimes/often/very often = 1), and having ever attempted suicide in prison (no = 0, yes = 1). Before analyzing the data all cases for which there was at least one missing observation were discarded.

All tests for significance were two-sided and p-values ≤ 0.05 were considered statistically significant and the significance level for removal variables from the models was set at p = 0.4. All analyses were conducted using the Stata software program, version 10.1 [19].

## Results

Of the 908 eligible male prisoners, 650 agreed to participate in the study by returning the questionnaire for a response rate of 71.6%. Table 1 shows demographic, detection, and lifestyle characteristics of respondent prisoners. The mean age was 39.8 years, more than half were married, one-third had at least one dependent child at home, had not attained compulsory education, and were unemployed at the time of prison entry. Two-thirds were current smokers, one-third had used illicit drugs over the course of their life and 20% of them admitted to having used illicit drugs behind bars. Less than half had been incarcerated for the first time, about two-thirds were permanently condemned, the mean time spent in prison was 6.8 years, and the mean length of sentence was 10 years.

**Table 1:** Demographic, detention, and lifestyle characteristics of respondent prisoners

Characteristic	**No**.	%
Age, years (623; 95.8)	39.8 ± 10.5^§^	
Marital status (628; 96.6)		
*Married*	363	57.8
*Other*	265	42.2
Having dependent children at home (638; 98.2)		
*None*	436	68.3
*Yes*	202	31.7
Educational attainment (631; 97.1)		
*No compulsory education*	202	32
*Compulsory education or some degree*	429	68
Occupational status before entering prison (643; 98.9)		
*Employed*	444	69
*Unemployed*	199	31
Cigarette smoking status (630; 96.9)		
*Past/Never smoker*	205	32.5
*Current smoker*	425	67.5
Daily amount of cigarettes smoked by current smokers (425; 65.4)	21.2 ± 12.3^§^	
First-time incarceration (638; 98.2)		
*No*	343	53.8
*Yes*	295	46.2
Time served in prison, years (588; 90.5)	6.8 ± 6.4^§^	
Detention status (621; 95.5)		
*Awaiting trial*	115	18.5
*Awaiting sentence*	102	16.4
*Serving sentence*	404	65.1
Length of sentence, years (397; 61.1)	10 ± 7.9^§^	
History of lifetime drugs use (619; 95.2)		
*No*	434	70.1
*Yes*	185	29.9
Type of drugs used prior to incarceration (173; 26.6)*		
*Cocaine*	132	75.9
*Marijuana/Hashish*	122	70.1
*Heroin*	61	35.1
*Barbiturates/Tranquillizers*	29	16.7
*Amphetamine*	25	14.4
*Other*	70	34.6
History of drugs use within prison (168; 25.8)		
*No*	133	79.2
*Yes*	35	20.8
Type of drugs used within prison (34; 5.2)*		
*Marijuana/Hashish*	18	52.9
*Barbiturates/Tranquillizers*	16	47.1
*Cocaine*	14	41.2
*Heroin*	8	23.5
*Ecstasy*	3	8.8
*Other*	9	26.5

In brackets the number and percentage of the total sample of 650 prisoners responding to the question

*Multiple responses allowed

^§ ^Mean ± standard deviation

### Self-reported health and quality of life

Table 2 shows the prisoners' perceived health status and quality of life. Overall, 31.6% and 43.5% of prisoners rated their health and perceived quality of life "poor". Compared to the time of prison entry, 60% reported that their health had worsened or greatly worsened and 35.1% were "extremely" concerned about their health. Multiple logistic regression analysis showed that older prisoners (OR = 1.06; 95% CI = 1.03-1.08), those with lower education level (OR = 0.54; 95% CI = 0.33-0.88), those experiencing negative feelings more frequently (OR = 4.13; 95% CI = 2.45-6.95), who attempted suicide within prison (OR = 2.48; 95% CI = 1.31-4.69), and who reported health problems on prison entry (OR = 2.54; 95% CI = 1.58-4.08) were significantly more likely to perceive a worse health status (*Model 1 in *Table 3).

**Table 2:** Prisoners' perceived health status and quality of life

	**No**.	%
In general, would you say your health is? (633; 97.4)		
*Poor*	200	31.6
*Good*	339	53.6
*Very good*	94	14.8
In general, would you say your quality of life is? (602; 92.6)		
*Poor*	262	43.5
*Good*	261	43.4
*Very good*	79	13.1
How would you rate, compared to prison entry, your health in general now? (623; 95.8)		
*Greatly worsened*	169	27.1
*Worsened*	205	32.9
*Unchanged*	218	35
*Improved*	19	3.1
*Greatly improved*	12	1.9
How much are you concerned about your health? (624; 96)		
*Extremely*	219	35.1
*Moderately*	228	36.5
*Not at all*	177	28.4

In brackets the number and percentage of the total sample of 650 prisoners responding to the question

**Table 3:** Multiple logistic regression analysis indicating association between several variables and the different outcomes

	Model 1. Self-rated health as poor			Model 2. Arranging a medical encounter more frequently		
	
	Log likelihood = -229.73, χ^2 ^= 116.44 (8 df), p <0.0001 470 observations			Log likelihood = -277.58, χ^2 ^= 79.88 (9 df), p <0.0001 468 observations		
**Variable**	**OR**	**95% CI**	**p**	**OR**	**95% CI**	**p**

Age, years	1.06	1.03-1.08	<0.0001	1.02	1.01-1.05	0.02
Marital status	*Backward elimination*			*Backward elimination*		
Attained compulsory education or more	0.54	0.33-0.88	0.014	*Backward elimination*		
Working before entering prison	*Backward elimination*			1.24	0.79-1.96	0.352
First time incarceration	*Backward elimination*			0.71	0.47-1.07	0.099
Time served in prison, years	0.97	0.93-1.01	0.103	*Backward elimination*		
Detention status						
*Awaiting trial*	1.00^§^	--	--	1.00^§^	--	--
*Awaiting sentence*	*Backward elimination*			1.41	0.72-2.75	0.315
*Serving sentence*	0.71	0.43-1.16	0.176	1.91	1.11-3.30	0.02
Feeling negative mood	4.13	2.45-6.95	<0.0001	2.00	1.31-3.05	0.001
Having ever attempted suicide within prison	2.48	1.31-4.69	0.005	1.48	0.75-2.92	0.261
Self-reported health problems on prison entry	2.54	1.58-4.08	<0.0001	3.81	2.32-6.25	<0.0001
Current smoker	*Backward elimination*			0.75	0.49-1.16	0.202
Having ever used drugs	1.26	0.74-2.14	0.393	*Backward elimination*		

^§^Reference category

### Self-reported health problems and access to health services within prison

Table 4 lists prisoners' self-reported current symptoms or diseases and reasons for arranging medical encounter. The rate of prisoners self-reporting health problems increased during the incarceration growing from 30% on prison entry to 82% at the time of questionnaire delivery. The top six health problems are dental health problems, arthritis or joint pain, eye problems, gastrointestinal diseases, emotional problems, and high blood pressure. Suicide was more frequently attempted within (13.6%) than outside the prison (7.9%). Furthermore, prisoners were afraid of possible STDs since over 60% of them rated a score ranging from 7 to 10. Prisoners consulted general practitioners about 6 times in the last year of incarceration while hospital admission was necessary for 11.4%. Consultations with general practitioners were significantly more frequent for older prisoners (OR = 1.02; 95% CI = 1.01-1.05), for those serving a sentence (OR = 1.91; 95% CI = 1.11-3.30), for those experiencing negative feelings more frequently (OR = 2.00; 95% CI = 1.31-3.05), and for those reporting health problems on prison entry (OR = 3.81; 95% CI = 2.32-6.25) (*Model 2 in *Table 3).

**Table 4:** Prisoners' self-reported symptoms or diseases and access to health services

Characteristic	**No**.	%
Self-reported health problems at prison entry (625; 96.2)		
*No*	439	70.2
*Yes*	186	29.8
Self-reported current symptoms or diseases (640; 98.5)		
*No*	116	18.1
*Yes*	524	81.9
Category of self-reported current symptoms or diseases (523; 80.5)*		
*Dental health problems*	293	56.1
*Arthritis or rheumatic pain*	210	40.2
*Eye problems*	199	38.1
*Gastro-intestinal diseases*	191	36.5
*Emotional problems (anxiety, depression)*	184	35.2
*High blood pressure*	129	24.7
*Cancer*	98	18.7
*Bone fractures*	92	17.6
*Liver diseases*	88	16.8
*Heart diseases*	87	16.6
*Emphysema or chronic bronchitis*	85	16.3
*Diabetes*	79	15.1
*Other*	54	10.3
Arranging a medical encounter in case of health complaints (625; 96.2)		
*Never*	53	8.5
*Rarely*	211	33.8
*Sometimes*	156	24.9
*Often*	132	21.1
*Very often*	73	11.7
Medical examination undergone during last year of incarceration (626; 96.3)		
*No*	87	13.9
*Yes*	539	86.1
Reason for medical examination during last year of incarceration (296; 45.5)*		
*Arthritis or rheumatic pain*	50	16.9
*Emotional problems (anxiety, depression)*	49	16.6
*Dental health problems*	43	14.5
*Check-up*	40	13.5
*Gastro-intestinal diseases*	32	10.8
*Heart diseases*	29	9.8
*Eye problems*	26	8.8
*Bone fractures*	24	8.1
*High blood pressure*	23	7.8
*Acute respiratory diseases*	22	7.4
*Other*	98	33.1
Hospital admission during last year of incarceration (599; 92.2)		
*No*	531	88.6
*Yes*	68	11.4
Having ever attempted suicide within prison (602; 92.6)		
*No*	520	86.4
*Yes*	82	13.6
Having ever attempted suicide outside prison (617; 94.9)		
*No*	568	92.1
*Yes*	49	7.9

In brackets the number and percentage of the total sample of 650 prisoners responding to the question

*Multiple responses allowed

### Participation to health promotion and social rehabilitative programs

Educational and health promotion programs, oriented towards the adoption of healthy behaviors in relation to alcohol consumption, STDs, smoking, and eating habits, are regularly provided within the prison healthcare system. Although all prisoners are invited to participate in these programs, only 9% were involved in programs smoking cessation/prevention, 8.9% in responsible alcohol consumption, 5.6% for prevention of STDs, and 6% to modify unhealthy eating habits. Moreover, almost half have participated in vocational training programs and 85% of them deemed such training "useful" but only 11.4% believed that it would allow them to find a new job after prison release.

## Discussion

To the best of our knowledge the present study is the first attempt to assess prisoners' self-reported health status in Italy. The results confirm previous studies that prisoners have a poor self-reported health status, high morbidity rate, and a frequent access to prison health services. It should be noted that we did not test the prison effect in determining the health status, the quality of life, and the access to health services, because we did not expect that structural and organizational differences between the selected prisons can play a role on these outcomes of interest. This is also supported by the results of a previous study conducted by some of us in the same four prisons with no significant differences among centers with regard the oral health status [20].

About 32% of prisoners rated their health as "poor", despite an average age of 40 years. In a study conducted on the Italian males general population, 1.9% of those aged 35-44 and 6.7% of all age groups rated their health as "poor" or "fair" and this percentage rises to 9.7% in Calabria. Moreover, the characteristics of the sample who perceived the worst health status were similar to those of the general population and it has been observed more frequently among those with a lower educational level and older [21]. These data clearly suggest that perceived health status is worse in the most disadvantaged subgroups. These findings are consistent with those reported in Australia where 28% of male prisoners rated their health as either "poor" or "fair" compared with 15% in the community [2]. Another study in the same area found that the perceived health was deemed as "poor" or "fair" by 24% and 30% of aboriginal and non-aboriginal male prisoners, respectively [22]. In the United States, half of newly admitted prisoners rated their health as "good", "fair", or "poor" [9], whereas in another study none of male prisoners rated their health as "poor", 20% as "fair", and 53% as "good" [23]. As expected, our findings that older age is a predictor of self-reported poor health is in accordance with other studies [16,23,24]. The finding that young prisoners are prone to evaluate their health more positively than those older is confirmed by Butler et al, who reported that 91% of offenders aged 15-24 in Australia evaluated their health as "excellent", "very good", or "good" [25]. Moreover, psychological conditions are also associated with perceived poor health and these factors are well documented risk factors for both poor health status and incarceration.

The prison system must be considered as a filter that selects and concentrates subjects with certain characteristics and trying to establish a relationship of causality about the effect of incarceration on health of prisoners may be misleading and controlling for confounders is very difficult. Other factors might simultaneously affect both incarceration and health and bias the apparent effect of incarceration. Instead, prison system should be regarded as an ally of public health, as an opportunity for providing treatment and education to individuals at risk and underserved. But prison can have long-term effects on prisoners' health. Schnittker et al [26] suggest that incarceration may have a negative effect on prisoners' health, in particular after release, since in custody they are unable to develop normal credentials and the distance and the time spent in prison negatively affect social integration, especially with their own family.

### Self-reported health problems and access to health services

A high prevalence of prisoners' self-reported diseases (82%), whereas lower values have been reported in the general population (13.1%) and in Calabria (15.7%) [21]. The top rated current health issue was dental health problems. This result is supported by the findings of a study, conducted by some of us, with only 2% of examined prisoners having no history of caries [20]. The authors also suggested the need for programs to improve oral health. Improving oral health can improve overall health [27]. Moreover, as expected considering the characteristics and the risk factors of the population surveyed, emotional and gastrointestinal problems were most frequently reported and this is consistent with previous results [2,9,16,28].

Among all demographic characteristics, only age was significantly associated with self-reported use of health services with older prisoners that were more likely to access to these services within prison. Not surprisingly, and according with previous studies, no effect has been found regarding other demographic characteristics [2,23,29,30]. It has been observed that prisoners seek care when ill and they use health services in prison 3 to 4 times more frequently than the general population and this use is linked to the high morbidity rate on entry, but this is not sufficient to explain the difference in health services utilization [31,32]. This inconsistency is particularly evident in our context in which both prisoners and general population have universal health care access. The high rate of health services use may also be explained by the fact that the therapy with methadone and buprenorphine is given for free to all prisoners with history of substance use as an effective treatment for opiate dependence and, hopefully, to reduce drug-related diseases and recidivism for prisoners.

Participation in health promotion programs was very low and this may reflect the lack of adequate programs offered to prisoners for the correction of unhealthy lifestyles. Newly admitted prisoners are advised by physician during examination, and effective prevention programmes are needed for people passing through the correctional system in Italy.

### Limitations

There are several potential limitations in this study. There is the possibility of a selection bias since almost 30% of prisoners did not participate and the mean age of the sample was slightly higher than that of the population of prisoners in the selected prisons, but this should not pose a threat to the generalizability to the overall prisoner population, since the group most prevalent in Italian prisons was between 30 and 39 years [33]. Moreover, caution is required in making cross-country comparisons of perceived health status, mainly because items assessing self-reported health status and quality of life were arranged on different level of responses. However, we believe that our results reflects the actual estimate of self-reported health. In fact, there are many alternative questionnaires assessing people's health and quality of life, each of them using different wording and different set of responses but the answers to the various self-reported health items are highly correlated [14]. Finally, misclassification may have been introduced by the use of self-reported history of health problems.

## Conclusions

The findings suggest that prisoners have a poor health status because of specific problems and care needs. However, prison health services are almost equivalent to those provided in the community since physicians act as general practitioners and most health problems are adequately addressed. But, the prison system appears to be a "bad" ally of public health considering that it seldom provides programs aimed at health promotion or facilitating social reintegration. Our findings give a cue for the provision of programs aimed at correcting risk behaviour and preventing the long-term effects of incarceration on prisoners' health.

## Competing interests

The authors declare that they have no competing interests.

## Authors' contributions

CGAN participated in the design of the study, data collection, statistical analysis, and interpretation of the data. DF participated in the statistical analysis, interpretation of the data, and wrote the article. GN participated in the design of the study and data collection. CP participated in the statistical analysis and interpretation of the data. IFA, the principal investigator, designed the study, was responsible for the data collection, statistical analysis, interpretation of the data, and wrote the article. All Authors read and approved the final manuscript.

## Pre-publication history

The pre-publication history for this paper can be accessed here:

http://www.biomedcentral.com/1471-2458/11/529/prepub
